# Supplementary material for: Vitamin D supplementation is associated with slower epigenetic aging
Source: GeroScience. 2022 May 13;44(3):1847–59. doi: 10.1007/s11357-022-00581-9 (PMC9213628; doi:10.1007/s11357-022-00581-9)
Supplement: Supplementary file 1 — Supplementary file1 (PDF 1259 KB) [file 11357_2022_581_MOESM1_ESM.pdf]

Vitamin D Supplementation is Associated with Slower Epigenetic Aging

Valentin Max Vetter[1, 2], Yasmine Sommerer[3], Christian Humberto Kalies[1], Dominik Spira[1], Lars Bertram[3, 4], Ilja Demuth[1, 5]

[1]Charité – Universitätsmedizin Berlin, corporate member of Freie Universität Berlin and Humboldt-Universität zu Berlin, Department of Endocrinology and Metabolic Diseases (including Division of Lipid Metabolism), Biology of Aging working group, Augustenburger Platz 1, 13353 Berlin, Germany

[2]Department of Psychology, Humboldt University Berlin, Berlin, Germany

[3]Lübeck Interdisciplinary Platform for Genome Analytics (LIGA), University of Lübeck, Lübeck, Germany

[4]Center for Lifespan Changes in Brain and Cognition (LCBC), Dept of Psychology, University of Oslo, Oslo, Norway

[5]Charité - Universitätsmedizin Berlin, BCRT - Berlin Institute of Health Center for Regenerative Therapies, Berlin, Germany

Corresponding author:

Ilja Demuth (Ph.D.)
Charité - Universitätsmedizin Berlin
Lipid Clinic at the Interdisciplinary Metabolism Center,
Biology of Aging Group
Augustenburger Platz 1
13353 Berlin
Email: ilja.demuth@charite.de
Phone: ++49 30 450 569 143
FAX: ++49 30 450 566 904

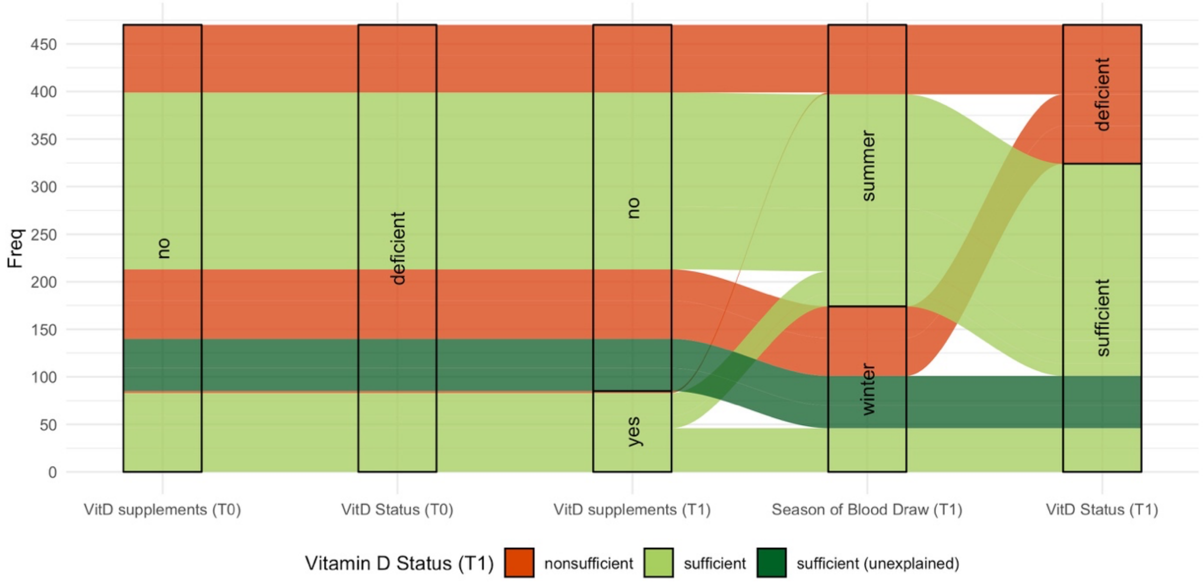

Supplementary Figure 1: Alluvial plot of participants that showed vitamin deficiency and no vitamin D supplementation at baseline. Many of the participants that had vitamin D level < 50nmol/L at baseline are vitamin D sufficient at follow-up. This transition can be explained for most participants by either start of vitamin D supplementation or blood draw during summer.

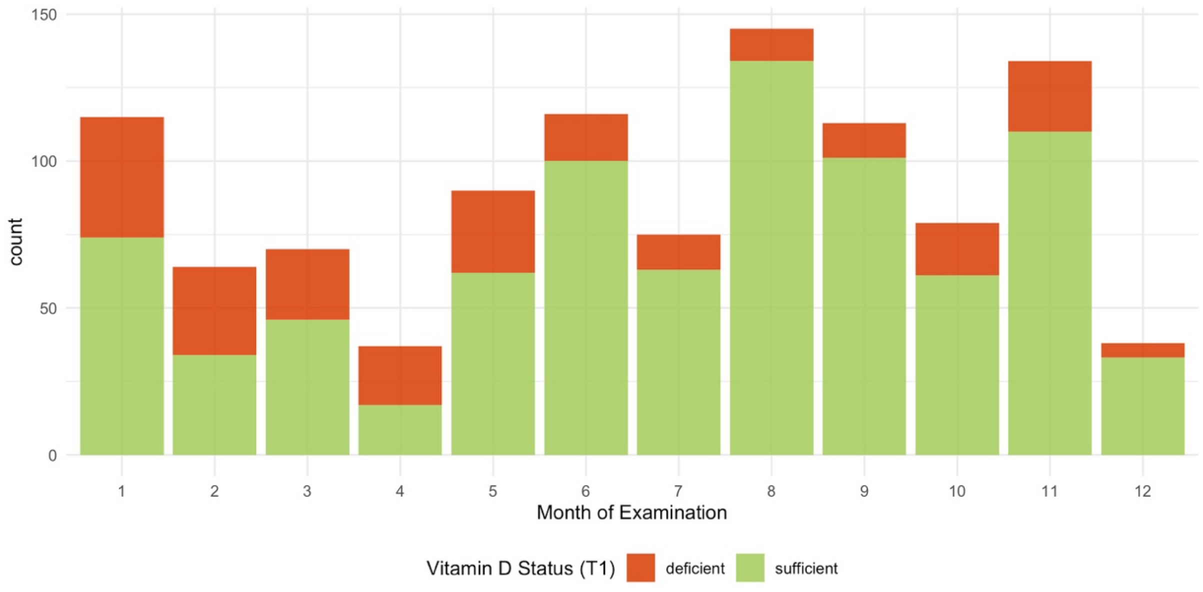

Supplementary Figure 2: Barplots of the frequency of vitamin D deficient participants stratified by month of blood draw.

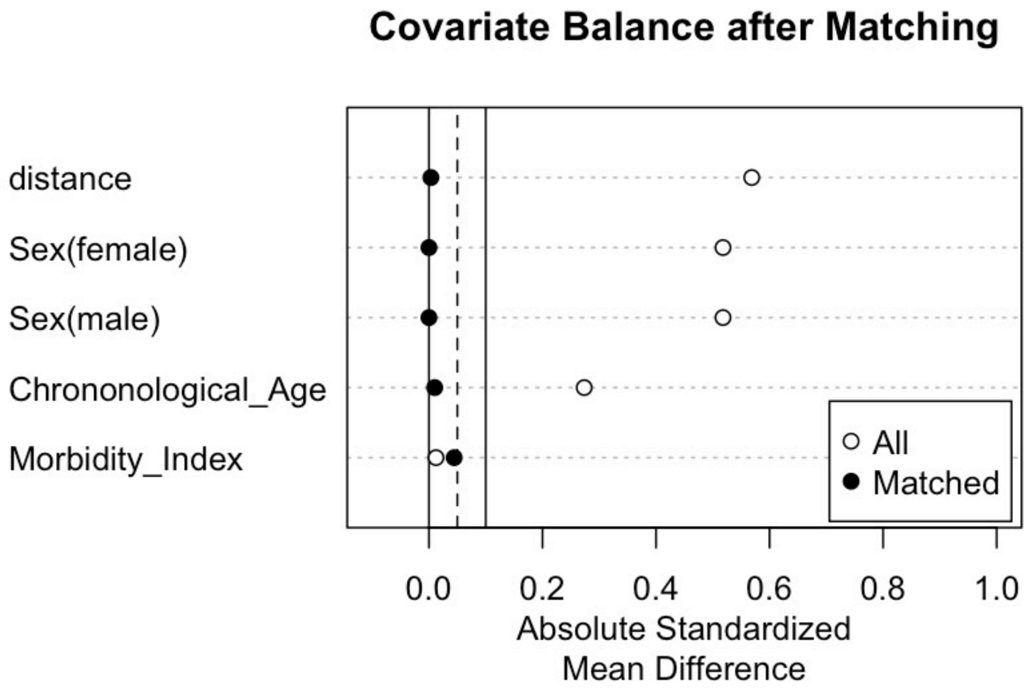

Supplementary Figure 3: Love plot of absolute standardized mean difference in covariates before and after matching of treated participants with vitamin D deficient participants without supplementation. Optimal pair matching was performed to identify the ideal control group for the participants that were vitamin D deficient at baseline and started vitamin D supplementation during the follow-up period.

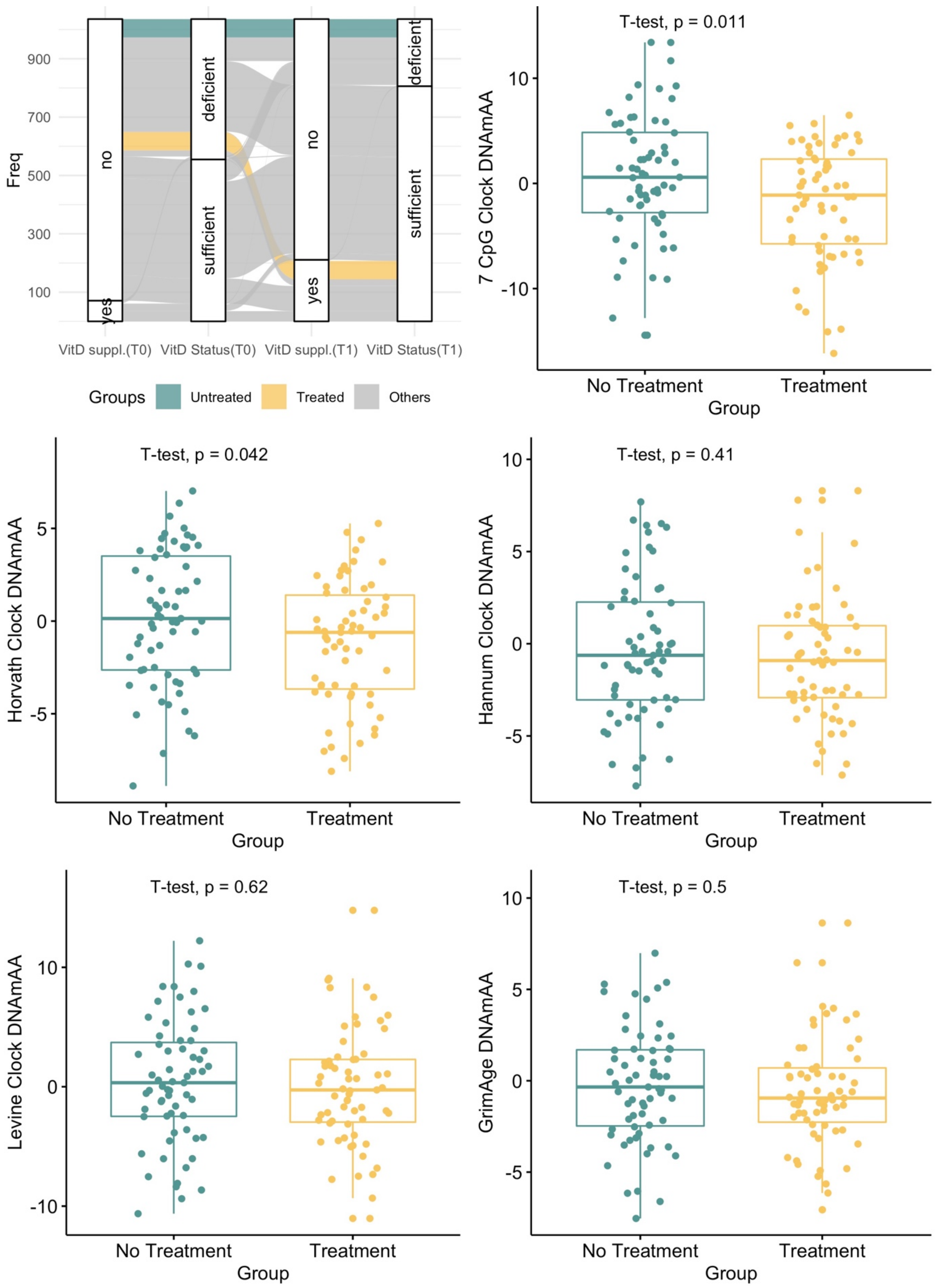

Supplementary Figure 4: Boxplots of DNAmAA by five different epigenetic clocks in the treated group (n=63) and control group (vitamin D sufficient at baseline and follow-up, n=63).

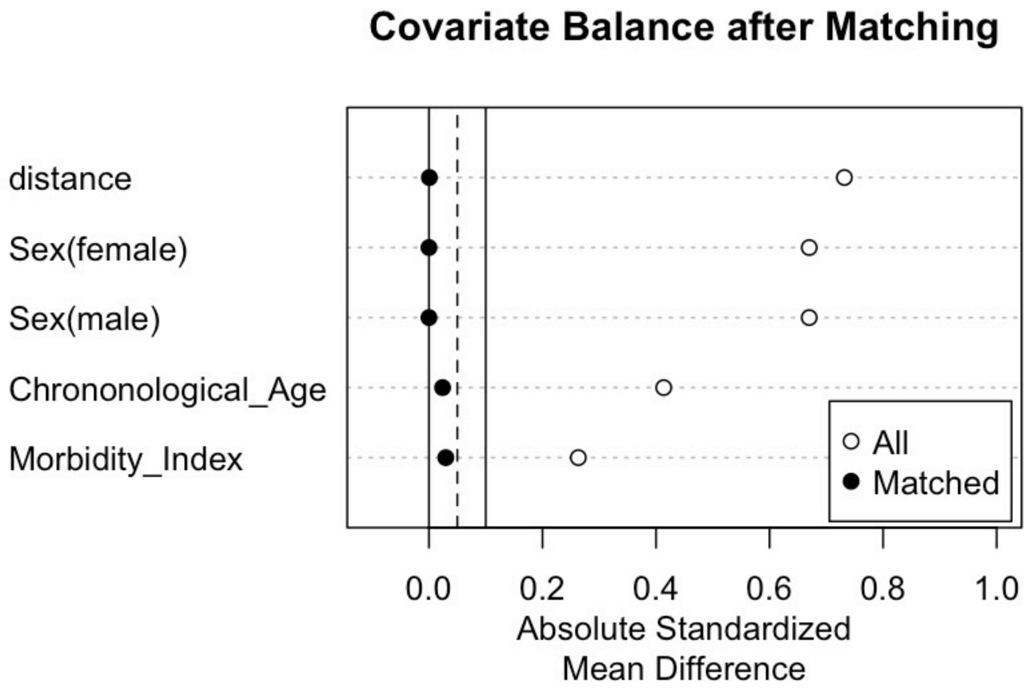

Supplementary Figure 5: Love plot of absolute standardized mean difference in covariates before and after matching of treated participants with vitamin D sufficient participants without supplementation. Optimal pair matching was performed to identify the ideal control group of "healthy" participants for the participants that were vitamin D deficient at baseline and started vitamin D supplementation during the follow-up period.

Supplementary Table 1: Sex-stratified cohort characteristics of the older participants at baseline examination (BASE-II). The t-test was used to assess the significance of the differences in means for continuous variables. Difference between categorical variables was determined by chi-square and goodness-of-fit test.

	female					male					
	n	Mean, %	SD	Min	Max	n	Mean, %	SD	Min	Max	p-value
Chronological Age	538	68.07	3.33	61.34	84.63	498	68.50	3.65	60.16	80.65	0.046
sex	538					498					
male	0	0.00				498	100.00				<0.001
female	538	100.00				0	0.00				
Vitamin D status	538					498					
deficient	253	47.03				228	45.78				0.735
sufficient	285	52.97				270	54.22				
Vitamin D supplementation	538					498					
no	484	89.96				481	96.59				<0.001
yes	54	10.04				17	3.41				
Season of blood draw	516					498					
winter	170	32.95				249	50.00				<0.001
summer	346	67.05				249	50.00				
Morbidity Index	433	1.11	1.21	0	10	434	1.28	1.34	0	7	0.050

Supplementary Table 2: Sex-stratified cohort characteristics of the older participants at follow-up examination (as part of the GendAge study). The t-test was used to assess the significance of the differences in means for continuous variables. Difference between categorical variables was determined by chi-square and goodness-of-fit test.

	female					male					
	n	mean	sd	min	max	n	mean	sd	min	max	p
Chronological Age	538	75.75	3.55	66.41	94.07	498	75.51	4.02	64.91	90.03	0.312
sex	538					498					<0.001
male	0	0.00				498	100.00				
female	538	100.00				0	0.00				
Vitamin D level (nmol/L)	538	72.82	27.78	8.75	218.20	498	68.07	26.68	8.75	200.3	0.005
Vitamin D status	538					498					0.555
deficient	115	21.38				115	23.09				
sufficient	423	78.62				383	76.91				
Vitamin D supplementation	538					498					<0.001
no	387	71.93				438	87.95				
yes	151	28.07				60	12.05				
Season of blood draw	538					498					0.824
winter	211	39.22				191	38.35				
summer	327	60.78				307	61.65				
Morbidity Index	464	1.33	1.49	0	9	438	1.44	1.61	0	9	0.282

Supplementary Table 3: Multiple linear regression analyses of DNAm age and DNAmAA on group (treated/control), season of blood draw, chronological age and sex (if applicable).

	Estimate	SE	p-value		n
Women and Men					
7-CpG DNAmAA	-2.55	0.99	0.0112	*	126
Horvath DNAmAA	-1.18	0.61	0.0557	.	126
Hannum DNAmAA	-0.46	0.62	0.4650		126
PhenoAge DNAmAA	-0.32	0.88	0.7177		126
GrimAge DNAmAA	-0.35	0.48	0.4659		126
Women					
7-CpG DNAmAA	-3.12	1.28	0.0165	*	86
Horvath DNAmAA	-0.57	0.79	0.4667		86
Hannum DNAmAA	-0.03	0.80	0.9730		86
PhenoAge DNAmAA	-0.34	1.12	0.7582		86
GrimAge DNAmAA	-0.28	0.55	0.6117		86
Men					
7-CpG DNAmAA	-1.49	1.48	0.3214		40
Horvath DNAmAA	-2.55	0.91	0.0077	**	40
Hannum DNAmAA	-1.22	0.96	0.2091		40
PhenoAge DNAmAA	-0.40	1.44	0.7811		40
GrimAge DNAmAA	-0.35	0.97	0,7236		40
